# COVID-19: In the Eye of the Cytokine Storm

**DOI:** 10.3389/fimmu.2020.558898

**Published:** 2020-09-24

**Authors:** Roberto de la Rica, Marcio Borges, Marta Gonzalez-Freire

**Affiliations:** ^1^Multidisciplinary Sepsis Group, Health Research Institute of the Balearic Islands (IdISBa), Palma de Mallorca, Spain; ^2^Vascular and Metabolic Pathologies, Health Research Institute of the Balearic Islands (IdISBa), Palma de Mallorca, Spain

**Keywords:** COVID-19, immunosenecence, inflammation, SARS-CoV-2, sepsis, aging

## Abstract

The dysregulated release of cytokines has been identified as one of the key factors behind poorer outcomes in COVID-19. This “cytokine storm” produces an excessive inflammatory and immune response, especially in the lungs, leading to acute respiratory distress (ARDS), pulmonary edema and multi-organ failure. Alleviating this inflammatory state is crucial to improve prognosis. Pro-inflammatory factors play a central role in COVID-19 severity, especially in patients with comorbidities. In these situations, an overactive, untreated immune response can be deadly, suggesting that mortality in COVID-19 cases is likely due to this virally driven hyperinflammation. Administering immunomodulators has not yielded conclusive improvements in other pathologies characterized by dysregulated inflammation such as sepsis, SARS-CoV-1, and MERS. The success of these drugs at reducing COVID-19-driven inflammation is still anecdotal and comes with serious risks. It is also imperative to screen the elderly for risk factors that predispose them to severe COVID-19. Immunosenescence and comorbidities should be taken into consideration. In this review, we summarize the latest data available about the role of the cytokine storm in COVID-19 disease severity as well as potential therapeutic approaches to ameliorate it. We also examine the role of inflammation in other diseases and conditions often comorbid with COVID-19, such as aging, sepsis, and pulmonary disorders. Finally, we identify gaps in our knowledge and suggest priorities for future research aimed at stratifying patients according to risk as well as personalizing therapies in the context of COVID19-driven hyperinflammation.

## Introduction

Accumulating evidence suggests that patients with severe COVID-19 develop a dysregulated release of cytokines also known as a “cytokine storm” or “cytokine storm syndrome.” The cytokine storm produces an excessive inflammatory and immune response, especially in the lungs, leading to acute respiratory distress (ARDS), pulmonary edema and multi-organ failure. In these situations, an overactive, untreated immune response can be deadly, suggesting that mortality in COVID-19 cases is likely due to this virally driven hyperinflammation. While the risk factors and phenotype profiles that cause otherwise healthy individuals to become critically ill still remain unknown, preliminary evidence suggests that other inflammatory processes such as aging or permanent lung damage may make one predisposed to a poorer prognosis. In this review, we summarize the latest data available about the role of the cytokine storm in COVID-19 disease severity as well as potential therapeutic approaches to ameliorate it. We also examine the role of inflammation in other diseases or conditions often comorbid with COVID-19, such as aging, sepsis, and pulmonary disorders. Finally, we identify gaps in our knowledge and suggest priorities for future research aimed at stratifying patients according to risk as well as personalizing therapies in the context of COVID19-driven hyperinflammation.

## Inflammation in Pathology and Disease

Inflammation is a vital phenomenon of a healthy immune response. However, dysregulated inflammation can result in severe damage, multisystemic organ dysfunction or even death. Rampant inflammation plays a central role in several pathologies such as sepsis, rheumatoid arthritis, respiratory diseases, cancer, and aging. Below we briefly review the role of inflammatory responses in several of these pathologies or conditions, which have been found to be aggravating factors in COVID-19.

### Inflammation and Aging

As we age the effectiveness of the innate and adaptative immune response declines, and this results in reduced protection against external pathogens, decreased ability to vaccination and increased susceptibility to infection, and limited repair capacity of damaged cells and tissues. This process is called “immunosenescence” (1) and it likely plays a central role in the age-related severity of COVID-19. Immunosenescence makes the innate immune response become more active, increasing the number of natural killer cells (NK) and releasing pro-inflammatory cytokines, such as Interleukin 6 (IL-6), Tumor Necrosis Alpha (TNFα), and C-reactive protein (CRP). In turn, this results in a chronic, low-grade inflammation, a phenomenon that has been termed as "inflammaging” (2). This chronic inflammation might contribute to biological aging and is a significant risk factor for age-related diseases, such as type 2 diabetes, Alzheimer's disease, hypertension, atherosclerosis, arthritis, hypertension, and cancer (2, 3). “Inflammaging” is a highly significant risk factor for both morbidity and mortality in the elderly. For example, Fabbri et al. (4) have shown that older people with a high baseline of IL-6 levels in combination with a faster increase in IL-6 levels over time have a significantly higher number of chronic diseases or multimorbidity as compared to those with high baseline levels but with a slower increase in IL-6 over time. IL-6 is a proinflammatory cytokine secreted by macrophages during the initial, acute phase of an inflammatory response. During this acute phase response, another downstream inflammatory marker, CRP is released in response to IL-6 (5). Both proteins are markers of systemic inflammation and predictors of mortality in older adults as well as in people with community-acquired pneumonia (6) which indicates that they could be used to identify individuals at higher risk of developing severe COVID-19 as well. In older people, muscle tissue has higher levels of these inflammatory cytokines, that together with a lack of physical activity, malnutrition, and hormonal dysregulation among other factors, may lead to the development of sarcopenia, the age-related loss of muscle mass and function that is one of the hallmarks of aging (3). This suggests that Clinical Frailty Indexes, which are rapid and already available in clinical practice, could be useful to screen for patients at risk of severe COVID-19.

### Inflammation and Sepsis

Sepsis is a life-threatening clinical process characterized by the dysregulation of homeostasis and the presence of a systemic inflammatory response syndrome. Sepsis is caused by an infection (from different types of pathogens, from bacteria to fungi and viruses) and leads to multiorgan dysfunction (7). Consequently, the dysregulated release of cytokines plays a central role in the syndrome's pathophysiology (8). Sepsis is strongly time-dependent, and it is known that the levels of inflammation biomarkers are prone to change abruptly. This makes it challenging to characterize cytokine profiles since they evolve rapidly as sepsis progresses. For example, it has been shown that IL-6, TNFα, and IL-10 levels peak within the first 2 h of the syndrome and then progressively decrease with time (9). Indeed, therapies based on blocking the proinflammatory action of cytokines such as TNFα and IL-1 have failed to improve sepsis outcomes in human trials because of the highly dynamic nature of these biomarkers, which have rapidly changing kinetic profiles (10, 11). Another confounding factor in characterizing sepsis is the heterogenous presentation of the syndrome, which varies depending on the type of infection (bacterial vs. viral, gram-negative vs. gram-positive), genetic polymorphisms and comorbidities. Recent advances in machine learning are overcoming this problem by enabling the simultaneous evaluation of extremely large volumes of data. For example, a recent article showed that sepsis patients can be categorized within 4 different phenotypes according to age and type of organ dysfunction, which could help stratify patients, predict their prognosis, and fine-tune therapeutic approaches in the near future (12).

Sepsis survivors are immunosuppressed, which makes them easy targets for viral infections such as COVID-19 (13). Likewise, nosocomial infections by polyresistant bacteria are a real threat for the critical COVID-19 patient, especially for those that require mechanical ventilation. Immunomodulatory therapies aimed at alleviating the cytokine storm originated by COVID-19 should be carefully designed in order to avoid putting these patients at a higher risk of bacterial or fungal sepsis.

### Inflammation and Respiratory Disease

Inflammation also plays a central role in respiratory diseases such as chronic obstructive pulmonary disease (COPD), asthma, and pulmonary fibrosis. In allergic asthma, epithelial cells respond to the presence of the allergen by producing cytokines such as IL-25 and IL-33 (14). In turn, these activate the TH2 response mediated by IL-4, IL-13, and IL-5 (15). IL-4 and IL-13 are important for regulating the production of IgE and the activation of macrophages (14), while IL-5 mediates eosinophilic inflammation (15, 16). COPD is a chronic inflammation of the lungs, which is usually triggered by long-term exposure to particulate matter or smoke. Consequently, consistently high levels of proinflammatory cytokines such as TNFα (17), IL-1 (18), or IL-6 (19) can be found in the sera of COPD patients. COPD may overlap with other respiratory conditions such as asthma or pneumonia. Exacerbation episodes due to environmental factors (pollen and pollution air levels) or respiratory viruses may cause an acute inflammatory response (20). For example IL-6 can be found at higher levels in the serum of patients going through an acute exacerbation episode (21). The accumulation of scar or fibrotic tissue in the lung may result in idiopathic pulmonary fibrosis. The process is largely mediated by TGF- beta (22). Extracellular matrix deposition in pulmonary fibrosis is parenchymal, and it has been proposed that it is the result of a “profibrotic cytokine storm” (14).

## Cytokine Storm in Coronavirus Diseases

Much can be learned about the role of inflammation in the course of the infections from previous respiratory coronaviruses. SARS-CoV-2, the severe acute respiratory disease coronavirus (SARS-CoV-1) and the Middle Eastern Respiratory Syndrome coronavirus (MERS-CoV) infect the lower respiratory airways and can cause severe pneumonia. During the first 9 days the patient shows flu-like symptoms such as cough and fever, often accompanied by diarrhea (23). During this phase, the virus replicates very fast and produces several proteins that are known to block interferon (IFN) responses (24). Histology studies show acute phase diffuse alveolar damage accompanied by edema, inflammatory infiltrate, and the formation of hyaline membrane (25). Shortly afterwards, viral titers in nasopharyngeal aspirates reach a peak and start decreasing. During this time, patients experience hypoxemia and high fever. By the third week about 20% will develop acute respiratory distress syndrome (ARDS) (23).

The production of chemokines by immune cells plays a central role in coronavirus-related hyper-inflammation. For example, infection of human monocyte-derived macrophages with SARS-CoV resulted in a very low production of IFN-γ but successfully induced the expression of CXCL10/IFN-inducible protein 10 and CCL2/monocyte chemotactic protein 1 (26). Chemokine upregulation was also observed after infection in dendritic cells (27). In serum, higher levels of pro-inflammatory cytokines and chemokines, through activation of Th1 cell-mediated immunity and hyper innate inflammatory responses, have been correlated to disease severity in SARS-CoV infections (28). Similarly, the “cytokine storm” responsible for the poor prognosis of MERS-CoV is controlled by T helpers 1 (Th1) mediators, and involves high levels of IFN as well as proinflammatory factors such as IL-1beta, IL-6, and IL-8, which are generated by airway epithelial cells (29). High serum levels of these cytokines are also indicative of severe MERS-CoV (30).

**Figure 1:**
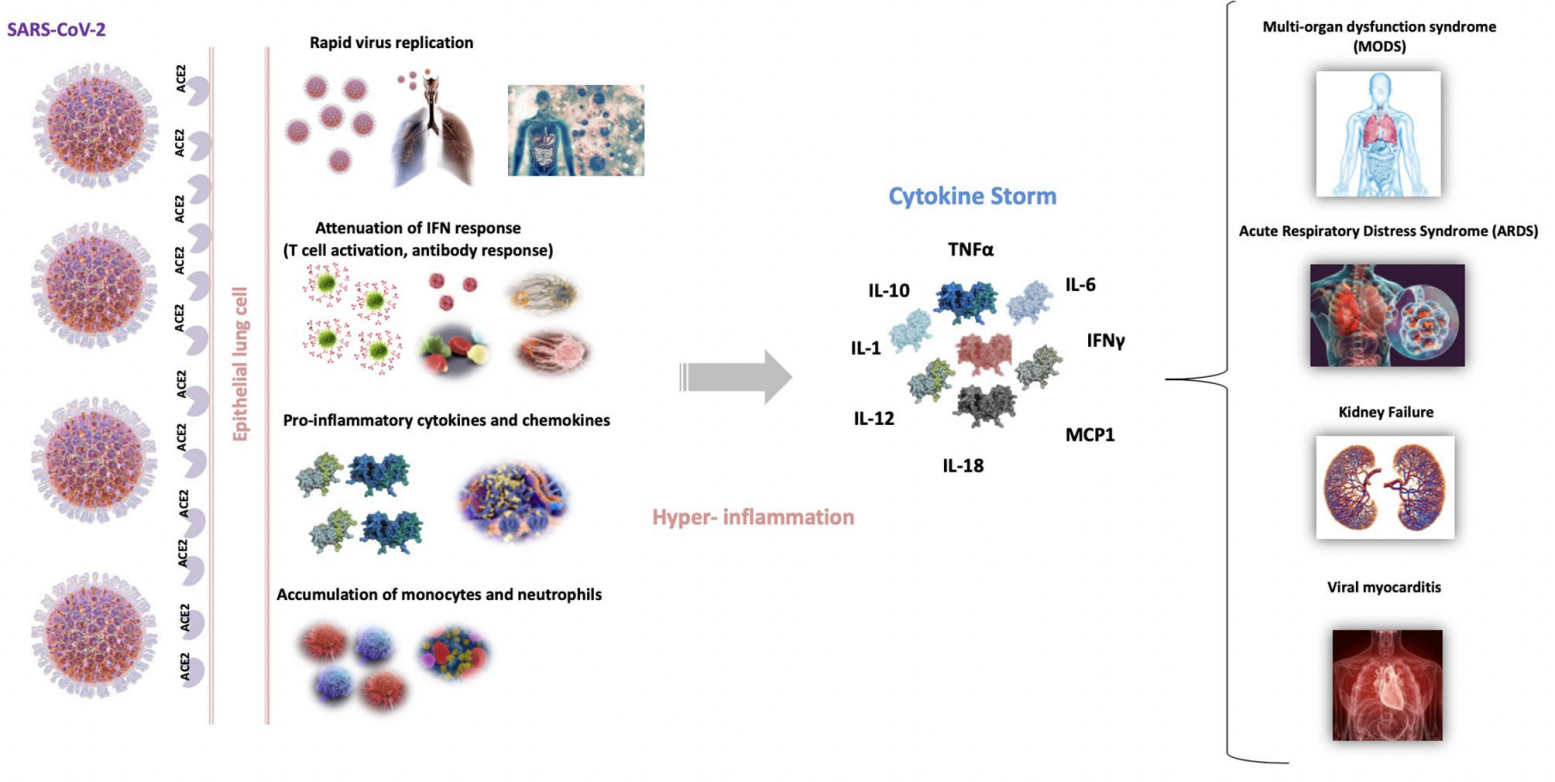
Schematic representation of the origin and repercussions of COVID-19 cytokine storm. Severe acute respiratory syndrome coronavirus 2 (SARS-CoV-2) into entry epithelial lung cells, binding to angiotensin-converting enzyme 2 receptor (ACE2). Th rapid viral replication in the first stages of the infection results in high proinflammatory state that attenuate and delay the IFN responses, which provokes an accumulation of pathogenic inflammatory macrophages. This, in turn, results in an even higher production of cytokines. This cytokine storm produces an excessive inflammatory and immune response, especially in the lungs, leading to ARDS, pulmonary edema, apoptosis of epithelial cells, vascular damage and multi-organ failure.

The origin of the dysregulated release of cytokines in these infections has been ascribed to diverse factors (31). It is assumed that the rapid viral replication in the first stages of the infection results in high proinflammatory responses. Furthermore, the virus generates high levels of proteins that are known to attenuate and delay IFN responses, which provokes an accumulation of pathogenic inflammatory monocyte-macrophages (24, 32). This, in turn, results in an even higher production of cytokines in the lungs (33). The consequences of this hyper-inflammation are also diverse, ranging from the dampening of T-cell responses, which leads to an even less controlled inflammatory response, to the apoptosis of epithelial cells, vascular damage, and ARDS (31) (Figure 1).

The dynamic interplay of factors involved in the cytokine storm generated by coronavirus infections makes it complicated to design therapies to halt its progression. For example, treatment with corticosteroids has been found to be mildly beneficial, not beneficial at all, or even deleterious in different studies (34–36). These disparate results show the complexity of the problem and the need to personalize timing and dosage for each particular case. Similarly, studies in macaques have shown that administering pegylated IFN-γ protects type 1 pneumocytes against SARS-Cov-1 infection when administered in the early stages of the infection. However, the same treatment had no effect on patients who were diagnosed at later stages, and therefore that had already progressed to severe MERS (37). These studies highlight the relevance of closely monitoring disease progression in order to maximize the benefits of therapies aimed at ameliorating coronavirus-induced hyperinflammation.

### Cytokine Storm in COVID-19

Table 1 summarizes the main findings published at the beginning of the pandemic (February 2020–April 2020) about COVID-19 and cytokine storm. The mortality of critically ill Chinese patients with SARS-CoV-2 pneumonia was between 50 and 62% (51, 52). The duration of terminal cases was usually 1–2 weeks after intensive care unit (ICU) admission. Older patients (>65 years) with higher SOFA score and ARDS were at increased risk of death (52). Several studies from countries that were first affected by the pandemic reported an increased prevalence of dysregulated immune responses in patients with COVID-19. This dysregulation is frequently accompanied by higher levels of inflammation or “hyperinflammation” and it is more likely to occur in elderly people with comorbidities, who have weaker immune functions and chronic inflammation as hypothesized above (53). It has been shown that aberrant pathogenic T cells and inflammatory monocytes are rapidly activated and produce a large number of cytokines, thus inducing this inflammatory storm (54) (Figure 1).

**Table 1:** Summary of the clinical studies with inflammatory markers in COVID19 patients.

**Population**	**Location**	^*^Age, Sex	**Laboratory markers**	**Cytokines and Chemokines**	**Treatment**	**References**
*N* = 8 (3 critically ill vs. 5 severe)	China	5 (2 mo−15) 75% (male)	↑WCC, ALT, PCT, CRP, D-dimer, LDH ↓LC =AST, CK	↑IL6, IFNγ = IL2, IL4	-Antiviral (100%) (oseltamivir virazole and/or interferon) -Antibiotic (62.5%) -Glucocorticoids (62.5%) -Traditional Chinese Medicine (50%) -Plasma infusion (25%) -IV immunoglobin therapy (50%)	(38)
*N* = 11 ICU severe patients	China	58 (26-72) 83% (male)	↑CRP, D-Dimer, LDH ↓LC = ALT, WBCC. CK	↑IL6, IL10 = IFNγ, IL2, IL4	-Antiviral (100%) -Antibiotic (100%) -Antifungal (91%) -Glucocorticoids (82%) -IV immunoglobin therapy (9.1%)	(39)
*N* = 21 (11 severe vs. 10 moderate)	China	56 (50-65) 81% (male)	↑WBCC, NC, AST, ALT. CK, LDH, D-Dimer, PCT, CRP, Ferritin ↓Albumin, LC = PC	↑IL6, IL10, IL2-R, TNFα = IL8	-Antiviral (82%) (oseltamivir and/or ganciclovir) -Antibiotic (100%) (moxifloxacin and/or cephalosporin) -Glucocorticoids (100%) (methylprednisolone)	(40)
*N* = 41 (13 ICU vs. 28 non-ICU)	China	49 73% (male)	↑WBCC, NC, Prothrombin Time, D-dimer, AST, ALT, Cardiac troponin I, PCT (initially normal, increased ICU with infection), CK, LDH ↓LC, Albumin, PC (not data on CRP)	↑IL1B, IL1RA, IL6, IL7, IL8, IL9, IL10, basic FGF, GCSF, GMCSF, IFNγ, IP10, MCP1, MIP1A, MIP1B, PDGF, TNFα, VEGF = IL5, IL12p70, IL15, Eotaxin, RANTES	-Antiviral (93%) (oseltamivir) -Antibiotic (100%) -Corticosteroids (22%)	(41)
*N* = 43 (15 severe vs. 28 mild)	China	54 (19-70) 60% (male)	↑CRP, Fibrinogen, ↑D-Dimer =WBCC, LC, AST, ALT, CK	↑ IL6	[Not available]	(42)
*N* = 48 (21 mild, 10 severe, 17 critically ill)	China	65 (47-83) 77% (male)	↑WBCC, Cardiac troponin I (mild and severe), AST (higher in critically ill and mild), ALT (higher in critically ill and mild), CK (higher in critically ill and mild), PCT ↓ LC, Cardiac troponin I (critically ill)	↑ IL6 (critically ill and mild)	[Not available]	(43)
*N* = 53 (34 severe vs. 19 moderate and) *N* = 8 healthy controls	China	^#^62 (22-78)	= WBCC, AST, ALT, CK ↓LC ↑ CRP, LDH, NC	↑IL1RA, IL6, IL10, IL18, CTACK, MIG, IFNγ, IP10 = IL2RA, MCP3, HGF, MIP1A, MCSF ↑IP10, MCP3, IL1RA (specially higher in severe vs moderate)	-Antiviral (38%) -Corticosteroids (30%)	(44)
*N* = 91 (9 severe vs. 82 mild)	China	50 41% (male)	↓WBCC, LC ↑D-Dimer, CK, CRP = AST, ALT, PCT	[Not available]	[Not available]	(45)
*N* = 94 (8 mild, 75 moderate, 11 severe)	China	^#^40 (1-78) 45% (male)	↑WBCC, NC, CRP, CK, LDH ↓ LC	↑ IL6	-Antiviral (49%) (IFN-α + lopinavir/ritonavir) -Antiviral (22%) (IFN-α + lopinavir/ritonavir+ ribavirin	(46)
*N* = 123 (21 severe vs. 102 mild)	China	52 (30-76) 66% (male)	↓ LC	↑IL6, IL10 =IL4, IL17, TNFα, IFNγ	[Not available]	(47)
*N* = 138 (36 ICU vs. 102 non-ICU)	China	56 (42-68) 54% (male)	↑WBCC, D-dimer, AST, ALT, CK, LDH, PCT, Cardiac troponin I ↓LC = PC	[No data available]	-Antiviral (90%) (oseltamivir) Antibiotic (100%) (moxifloxacin [64.4%]; ceftriaxone [24.6%]; azithromycin, [18.1%]) -Glucocorticoids (45%)	(48)
*N* = 150 (68 death vs. 82 discharged)	China	58.5 (15-81) 68.5% (male)	↑WBCC, MB, AST, ALT, BUN, CK, LDH, Cardiac troponin I, CRP, Ferritin ↓ LC, PC	↑ IL6	-Antiviral (58%) -Antibiotic (96%) -Antifungal (12%) -Glucocorticoids (37%)	(47)
*N* = 452 (286 severe vs. 166 non severe)	China	58 (47-67) 52% (male)	↑Leukocytes, NLR, PCT, ESR, Ferritin, CRP ↓ LC	↑ IL6, IL2-R, IL8, IL10, TNFα	[No data available]	(49)
*N* = 1,099 (173 severe vs. 926 non-severe)	China	47 (35-58) 58% (male)	↑CRP ↓WBCC, LC = D-Dimer, AST, ALT	[Not available]	-Antiviral (36%) (oseltamivir) -Antibiotic (58%) -Antifungal (3%) -Glucocorticoids (19%)	(50)

*WBCC, White blood cell count; ALT, Alanine aminotransferase; PCT, procalcitonin; CRP, C-reactive protein; LDH, lactate dehydrogenase; LC, lymphocyte count; AST, Aspartate aminotransferase; CK, creatine kinase; IV, intravenous; PC, platelet count; NC, neutrophil count; MB, myoglobin; BUN, blood urea nitrogen; ESR, Erythrocyte sedimentation rate; NLR, Neutrophil-to-lymphocyte ratio*.

A recent paper published in Lancet was the first to report the epidemiological, clinical, laboratory, and radiological characteristics, treatment, and clinical outcomes of 41 laboratory-confirmed cases infected with SARS-CoV-2 (41). In this study the authors showed that severe COVID-19 patients who developed ARDS due to higher inflammation were more likely to die. Both, Th1 pro-inflammatory cytokines and Th2 anti-inflammatory cytokines were higher in COVID-19 patients. Of note, those with higher levels of Th1 cytokines required ICU admission, suggesting that the cytokine storm was associated with disease severity and consequently with a worse prognosis.

Interestingly Quin et al. (49) found that the severe group with COVID-19 had higher neutrophil count and a lower number of lymphocytes, inducing a cytokine storm in the body and damage in the lungs and heart, among other organs. It is known that the increase of neutrophil-to-lymphocyte ratio (NLR), is a marker of systemic inflammation and infection that could be used as a predictor of bacterial infection, including pneumonia. The increase of NLR in this study is consistent with the findings from Wang et al. (48). These authors also found a relevant increase in the levels of procalcitonin (PCT), another marker of infection, used regularly in the clinic as a marker to aid in the diagnosis of bacterial infections and to guide antibiotic therapy (55, 56). These findings are also consistent with the recent report from Huang et al. (41).

Chen et al. (40) found that the SARS-CoV-2 infection induced a cytokine storm (increase in IL-6, IL-2R, IL-10, and TNFα) and lymphopenia, a decrease in CD4^+^ and CD8^+^T cells, as well as suppressed IFN-γ production by CD4^+^T cells, which might be correlated with disease severity in COVID-19.

Zheng et al. (57) found no statistical differences in IL-6 and TNF-α plasma concentrations in mild or severe COVID-19 patients. However, they showed that elevated exhaustion levels and reduced functional diversity of T cells in peripheral blood may also predict severe progression in COVID-19 patients. A study in 30 COVID-19 patients found that the platelet to lymphocyte ratio (PLR) was associated with a poorer prognosis and a longer than average hospitalization time. The authors suggest that the PLR of patients could be a proxy of the cytokine storm, since the recruitment of neutrophils and other inflammatory cells to the site of injury plays a crucial role in the inflammatory response, providing a new inflammation index to monitor patients with COVID-19 (58).

Another recent study reported that the serum SARS-CoV-2 viral load (RNAaemia) is strongly associated with the levels of IL-6 in COVID-19 patients (43). The levels of IL-6 were 10-fold higher in critically ill patients compared to severe patients. This study strongly suggests that IL-6 is a promising prognosis biomarker and therapeutic target in critically ill COVID-19 patients. Similarly, another study found that the combination of IL-6 and D-Dimer measurements had the highest specificity and sensitivity for early prediction of the severity of COVID-19 patients (~94%), and that they were also useful to track pneumonia development (42). Levels of lactate dehydrogenase (LDH) and creatine kinase (CK) have been associated with viral mRNA elimination, suggesting that a constitutive decrease of LDH or CK levels probably predict a favorable recovery response for COVID-19 patients (46).

The serial detection of IFN-γ-induced protein 10 (IP-10), monocyte chemotactic protein-3 (MCP-3) and IL-1ra in 14 severe cases showed that the continuous high levels of these cytokines were associated with disease deterioration and fatal outcome (44). The authors suggest that the combination of these cytokines are independent predictors for the progression of COVID-19.

Several studies have also reported that the activation of coagulation pathways (i.e., increased D-dimer, a marker of thromboembolism), during the immune response to COVID-19 might also lead to an overproduction of proinflammatory cytokines leading to multiorgan injury and death (59).

In summary, early reports show a correlation between the cytokine storm syndrome and severity leading to a poor prognosis in COVID-19.

Below we also review early therapeutic attempts at relieving this hyperinflammatory state with available drugs at the time of the pandemic.

## Re-purposed FDA Drugs That Have Been Used to Treat Cytokine Storm in COVID-19 Patients

Currently, the preferred therapeutic approach to COVID-19 seems to be a multimodal treatment combining antibiotics, antiviral (i.e., remdesivir, favipiravir, lopinavir/ritonavir, etc.), and anti-inflammatory drugs with support therapies for the respective organic failures (60, 61). Antiviral treatment is important to decrease viral load and replication, decreasing RNAemia and consequently the inflammatory stimulus. The main immunomodulatory agents used for COVID-19 at the beginning of the pandemic are summarized in Table 2. Later on, some of these drugs have demonstrated to have no effect on survival and prognosis of the disease.

**Table 2:** Potential immunomodulators used for COVID-19 disease at the beginning of the pandemic.

**Drug**	**Clinical use**
Azitromicine	Antibacterial, Immunomodulator
Steroids	Anti-inflammatory, Septic Shock ARDS
IFN-β 1b	Rheumatic Diseases Hepatitis C
Tocilizumab	Anti-IL6R Rheumatic Diseases, Cytokine Storm
Sarilumab	Release Syndrome in CAR-T cells
Baricitinib	JAK inhibitor, Rheumatic Diseases
Anankinra	IL-1R antagonist Autoimmune Diseases
Convalescent Serum	Treatment of SARS and MERS
Immunoglobulins	Autoimmune Diseases
Nitric oxide	ARDS Lung Hypertension
Remdesivir, Favipiravir, Lopinavir/Ritonavir	Antiviral treatment

*ARDS, acute respiratory distress syndrome; JAK, Janus tyrosine kinase; IL-1R, interleukin 1 receptor; IL-6R, interleukin 6 receptor; CAR-T cells, Chimeric antigen receptor T*.

Emerging evidence indicates that there are potential benefits when managing the cytokine storm in COVID-19 patients by administering steroids, IL-6/IL-6-receptor (IL-6R) blocking antibodies, TNF inhibitors, IL-1 antagonists, and Janus kinase inhibitor (JAK) inhibitors (62). Steroids inhibit the synthesis of various cytokines (IL1-8, TNFα, IFNg, GM-CSF) produced by an array of different cells (macrophages, monocytes, lymphocytes, endothelial, or epithelial). Steroids act through a variety of mechanisms, including the inhibition of transcription, by preventing protein translation and destroying mRNA, by inhibiting the synthesis of cytokine receptors and activating transcriptional factors such as AP-1 or NF-kB, and by genetic dispersion of adhesion molecules (ICAM-1). Glucocorticoids may also modulate inflammation-mediated lung injury. Recently, a controlled, open-label trial evaluating the potential use of oral or intravenous dexamethasone for critically ill COVID-19 showed a decrease in mortality among those who were receiving either invasive mechanical ventilation or oxygen alone at randomization but not among those receiving no respiratory support (63).

Tocilizumab is an FDA-approved immunosuppressive drug targeting IL-6R that is commonly used for the treatment of rheumatoid arthritis (RA) (64). Tocilizumab has also been used to manage cytokine release syndrome (CRS) in patients receiving chimeric antigen receptor (CAR) T cell therapy (65). In COVID-19 patients, Tocilizumab has been shown to improve the clinical symptoms by reducing the inflammation and decreasing severity (54). Several drugs such as Sarilumab (IL-6R antagonist), anakinra (IL-1 receptor antagonist), Baricitinib, Fedratinib, and ruxolitinib (JAK inhibitors) are under study at the moment in several phase II/III clinical trials around the world.

Wu and Yan (66) suggest that in COVID-19 patients the FDA approved JAK2 inhibitor Fedratinib, in combination with anti-viral drugs, could be used to reduce the mortality associated with hyperinflammation by suppressing the production of several Th17 cytokines (i.e., IL1b and TNFalpha, IL21, IL22, IL17) and the formation of pulmonary edema. It has also been suggested that modulators targeting the cytokine IP-10 are a promising therapeutic strategy in the treatment of the acute phase of ARDS since they could ameliorate acute lung injury (44).

Hydroxychloroquine (HCQ), an anti-malarial drug which exhibits an antiviral effect similar to that of chloroquine, could mitigate the severe progression of COVID-19, inhibiting the cytokine storm by suppressing T cell activation (67). HCQ has been shown to decrease the production of IFN, TNF, IL-6, and IL-1 and promote autophagic inhibition. However, recent results from a clinical trial have shown that the use of hydroxychloroquine, alone or with azithromycin, did not improve clinical status of COVID-19 patients compared to standard care (68).

Azithromycin is an antibacterial agent (macrolide) that is also being used to treat COVID-19 patients in combination with other drugs such as hydroxychloroquine. It has a well-known immunomodulatory effect and could be an important element of multimodal treatments, even in seriously ill patients (69).

Blood purification, a treatment in which a patient's blood is passed through a device to remove waste products, and toxins, is a promising therapy in reducing the cytokine storm that occurs as a late complication of critically ill COVID-19 patients, although the small cohort (*n* = 3) merits more investigation (70). Administering convalescent plasma is a promising alternative that has been used with notable success in SARS-Cov1, MERS, or Ebola patients. The plasma must be collected from recovered patients who can donate blood, do not show any symptoms for more than 14 days and have yielded negative results on Covid-19 tests. The small clinical experience with using this method for treating COVID-19 is encouraging, although it will require a thorough examination and more contrasted data through clinical trials (71). Another alternative is the use of therapeutic plasma exchange for fulminant COVID-19 patients. The main arguments for using this treatment would be to removing cytokines, stabilizing endothelial membranes and resetting the hypercoagulable state.

While therapeutic outcomes are rather anecdotal at the moment, several ongoing large-scale clinical trials will surely shed more light into the validity of these and other therapeutic approaches aimed at ameliorating the cytokine storm in COVID 19 patients. However, judging from the results obtained from previous SARS cases, the success of these therapies will likely be intimately tied to timing and dosage, due to the highly dynamic nature of the inflammatory process. No proven benefits of immunomodulatory treatments in severe COVID-19 infections are known at the moment. Additional risks such as a higher incidence of opportunistic infections or reactivations of infections such as hepatitis b under treatment with Tociluzimab should be taken into account. Therefore, the use of these immunomodulators in COVID-19 must be evaluated very critically. Finally, by the time this manuscript was written, only the treatment with Remdesivir has shown some clinical improvement (i.e., shortening the time to recovery) in COVID-19 patients (72, 73).

## Conclusion

To date, no available FDA drug or therapy has demonstrated 100% efficacy for patients with COVID-19. The high percentage of critically ill patients in the COVID-19 pandemic has forced some ICUs to take desperate measures. In this context, it is imperative to identify biomarkers for predicting disease severity and prognosis in order to make more efficient choices regarding the use of limited resources in ICUs as to avoid their oversaturation. According to early reports depicted in Table 1, increased levels of inflammatory markers such as D-dimer, CRP, IL-6, and CK, together with a reduction in lymphocyte counts, and increased ferritin levels are common in COVID-19 patients and have been associated with severe stages of COVID-19. Close monitoring of these biomarkers could reveal the evolution from mild to severe COVID-19 and avoid poor outcomes in future cases.

There is a need to better understand the disease, its pathophysiology, temporal evolution, prognostic clinical and analytical parameters, the immunity (or not) generated, and the real impact of the proposed treatments that are being evaluated in different clinical trials at this moment. Lessons learnt from other hyperinflammatory syndromes such as sepsis and the cytokine storm responsible for the poor outcomes in SARS and MERS show a strong time dependence between cytokine levels and disease progression. We propose that measuring inflammation biomarkers frequently over time is the best strategy to characterize the evolution of the cytokine storm, since it provides a personalized biomarker profile for each patient. In turn, this could be used for guiding the timing and dosage of anti-inflammatory treatments, as well as to assess their effectiveness. Point-of-care diagnostic devices will likely be crucial to enable these kinetic biomarker measurements without collapsing central diagnosis laboratories (74–77).

**Figure 2:**
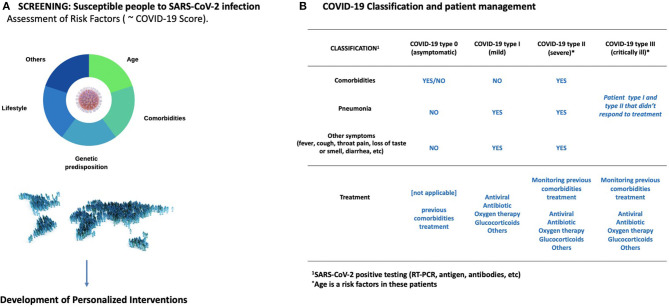
Prevention and management COVID-19**. (A)** There is an urgent need to improve our understanding on the phenotype profiles behind the progress from mild to severe or critical COVID-19. This includes analyzing the different risk factors as well as circulating biomarkers. In turn, this could lead to personalized therapies with reduced side effects. It is also imperative to screen the elderly for risk factors that predispose them to severe COVID-19. Immunosenescence and comorbidities should be taken into consideration **(B)** An operative classification to screen COVID-19 patients is showed in this panel. Age is a risk factor specially in the severe and critically ill patients.

Young individuals or people with no associated morbidities typically have mild symptoms or remain asymptomatic, while the elderly experience substantially more severe symptoms and lethality. Prevention and screening strategies should be implemented to manage COVID-19 disease (Figure 2). Older patients (>60 years) with comorbidities or chronic medical conditions and ARDS are at increased risk of death from COVID-19. This is likely due to immunosenescence and increased frailty associated with aging, that lead to a loss of function and fitness. It is noteworthy that even after a SARS-CoV-2 vaccine is developed and available as a prevention strategy, it might not provide full protection to the elderly due to this decline in immune function. Evidence from other diseases such as H1N1 has shown that the effectiveness of the influenza vaccine differs annually due to mutations in the virus. Similar scenarios can be expected with the COVID-19 pandemic, which makes it prescient to study other preventive strategies such as screening the most vulnerable population. Clinical frailty indexes could provide a simple method to identify these patients. This could be supplemented with measurements of inflammation biomarkers such as IL-6, which are, to date, the most consistent and reliable risk markers for adverse health outcomes in older people (3). Also, older COVID-19 patients who become critically ill often have cardiovascular, metabolic and/or kidney diseases and/or cancer, among other complications. Therefore, close monitoring of previous (multi)morbidities during hospitalization is of vital importance in order to discern their role in COVID-19 disease progression. Recent advances in Artificial Intelligence (AI) could make possible a holistic view of the COVID-19 disease, as the AI can combine and analyze all the patient's clinical history data with biomarker measurements, frailty scores, and therapeutic interventions, all within seconds. Once trained, the AI could also be used to identify patients at risk of severe or critical COVID-19 that require special care.

Finally, there is an urgent need to develop rehabilitation treatments for COVID19 survivors. Patients with respiratory symptoms who survive the virus might be probably left with chronic problems, such as lung fibrosis, lower lung function, and kidney damage (78, 79). Interventions such as oxygen therapy, physical activity and others focused on the mitigation of these chronic conditions is crucial as well. Similarly, a long-term study of inflammation markers may shed new light on the impact of the cytokine storm in COVID19 survivors.

## Author Contributions

RR, MB and MG-F contributed to develop the content of the manuscript and wrote the manuscript. All authors contributed to the article and approved the submitted version.

## Conflict of Interest

The authors declare that the research was conducted in the absence of any commercial or financial relationships that could be construed as a potential conflict of interest.
